# Focused shockwave induced blood-brain barrier opening and transfection

**DOI:** 10.1038/s41598-018-20672-y

**Published:** 2018-02-02

**Authors:** Yi Kung, Chiang Lan, Ming-Yen Hsiao, Ming-Kuan Sun, Yi-Hua Hsu, Abel P.-H. Huang, Wei-Hao Liao, Hao-Li Liu, Claude Inserra, Wen-Shiang Chen

**Affiliations:** 1 Department of Physical Medicine and Rehabilitation, National Taiwan University Hospital & National Taiwan University College of Medicine, Taipei city, Taiwan; 2 Faculty of Medicine, National Yang-Ming University, Taipei city, Taiwan; 3 Department of Surgery, National Taiwan University Hospital, Taipei city, Taiwan; 4 Department of Electrical Engineering, Chang Gung University, Taoyuan, Taiwan; 5 LabTAU, INSERM, Centre Léon Bérard, Université Lyon 1, Univ Lyon, F-69003, Lyon, France

## Abstract

Despite extensive efforts in recent years, the blood-brain barrier (BBB) remains a significant obstacle for drug delivery. This study proposes using a clinical extracorporeal shockwave instrument to open the BBB, combined with a laser assisted bi-axial locating platform to achieve non-invasive, controllable-focus and reversible BBB opening in the brains of rats. Under shockwave treatment with an intensity level of 5 (P^–^9.79 MPa, energy flux density (EFD) 0.21 mJ/mm^2^) and a pulse repetition frequency of 5 Hz, the BBB could be opened after 50 shocks without the use of an ultrasound contrast agent. With the proposed method, the BBB opening can be precisely controlled in terms of depth, size and location. Moreover, a shockwave based gene transfection was demonstrated using a luciferase gene.

## Introduction

The blood-brain barrier (BBB) remains a crucial obstacle in the delivery of medication into the parenchyma and central nervous system (CNS). The BBB is a physical barrier made of brain capillary endothelial cells, tight junctions, basement membranes, adjoining pericytes, astrocytes and microglia. Only molecules with a mass less than 400–500 Dalton are able to cross the BBB in pharmacologically significant amounts, raising significant obstacles to treating CNS related disorders^1,2^. Currently, more than 20 million patients worldwide suffer from CNS-related disorders, and further attempts are needed to open the BBB in a non-invasive, localized, and transient manner to allow for the delivery of therapeutic agents directly into the brain^3–5^.

Progress in molecular biology has provided a better understanding of the BBB, particularly under different biological and pathological conditions, facilitating drug delivery through chemical therapy, such as extracting cholesterol from brain capillary endothelial cell membranes to open tight junctions using cyclodextrin^6^, binding neuron cell acetylcholine receptors by peptide modification^7,8^, upregulating chemokines to open tight junctions by virus^9,10^, causing a Trojan Horse effect by macrophage^11,12^, physical treatments to trigger transcytosis, transendothelial openings, partial opening of tight junctions by ultrasound^13,14^, increasing BBB permeability via microwave-based thermal effects^15^, opening tight junctions via protein kinase C signaling, and tight junction protein translocation by electromagnetic fields^16,17^.

However, such chemistry and biology-based practices provide only limited drug delivery and short-duration response across the BBB, and produce side effects which can negatively impact later quality of life^18,19^. In contrast, high-intensity focused ultrasound (HIFU) combined with microbubbles presents a noninvasive method to locally and transiently disrupt the BBB at discrete targets^20,21^. However, ultrasound therapy can produce thermal damage during sonication, and transducing depths are limited at higher frequencies^22^.

The current study tests the feasibility of using a commercial extracorporeal shockwave device, originally designed for the treatment of various soft tissue pathologies, to enhance the CNS-blood permeability^23^. Shockwaves are well known to produce cavitation^24^, which is believed to be the major mechanism responsible for opening the BBB by HIFU^25,26^. Furthermore, focused shockwaves operate at a lower frequency than ultrasound, thus offering better penetration. Suitable shockwave devices are commercially available and thus no complicated HIFU devices need be built for CNS applications.

## Material and Methods

### Bio- and chemical materials

The study proposal was approved by the ethics committee of the Laboratory Animal Center at National Taiwan University College of Medicine (approvals No. 20170091 for the use of rats), and adhered to the experimental animal care guidelines. All rats (adult Sprague Dawley (SD) rats, 8 weeks) were purchased from the National Laboratory Animal Center (Taipei City, Taiwan), and were divided into three groups receiving shockwave (96 rats), microbubble (20 rats), and gene-transfection (15 rats) treatment between 9 to 10 weeks of age (a total of 131 male rats). pCI-Neo-Luc+ (7187 bp, around 26591 Da), purified by Qiagen Mega endotoxin free, was acquired from the Biomedical Resource Core of the First Core Laboratory, College of Medicine, National Taiwan University (Taipei, Taiwan). D-Luciferin Firefly potassium salt was purchased from Biosynth AG (Lake Constance, Switzerland). 150 mM NaCl, sterile-filtered by 0.22 μm PES membrane (Millipore syringe filter) was purchased from Polyplus-transfection (Illkirch, France). Dulbecco’s phosphate-buffered saline (DPBS 10×, Gibco) was purchased from Thermo Fisher Scientific Inc. (Waltham, US). Forane (Isoflurane) was purchased from Aesica Queenborough Ltd. (Queenborough, UK). Isotonic sodium chloride solution (0.9%) was purchased from Taiwan Biotech Co., LTD. (Taoyuan, Taiwan). A peroxidase *in situ* apoptosis detection kit (TUNEL S7100, ApopTag) was purchased from Merck KGaA (Darmstadt, Germany). Anti-Luciferase antibody produced in rabbits was purchased from Sigma-Aldrich Co. LLC. (St. Louis, US). SonoVue was purchased from Diagnostics Inc., (Milano, Italy). Ultrasound coupling gel (CG955, sonic resistance: 1.55 ± 0.05 MRayl, pH 7.0 ± 0.05) was purchased from Ceyotek (Chiayi City, Taiwan). All materials and their derivatives were processed at biotechnology grade.

### Instruments and devices

The shockwave device (PiezoWave) was purchased from Richard Wolf GmbH (Knittlingen, Germany)*. In vivo* imaging system spectrum (IVIS) and an XGI-8 gas anesthesia system (PerkinElmer, Waltham, US) were equipped in the Laboratory Animal Center at National Taiwan University College of Medicine. A fixed stage microscope (BX51) was purchased from Olympus Co. (Tokyo, Japan). A digital microscope camera system (RT-KE Color 3-shot, SPOT) and its software (SPOT advanced) were purchased from Diagnostic Instruments, Inc. (Sterling Heights, US).

### Localization of shockwave probe

**Figure 1:**
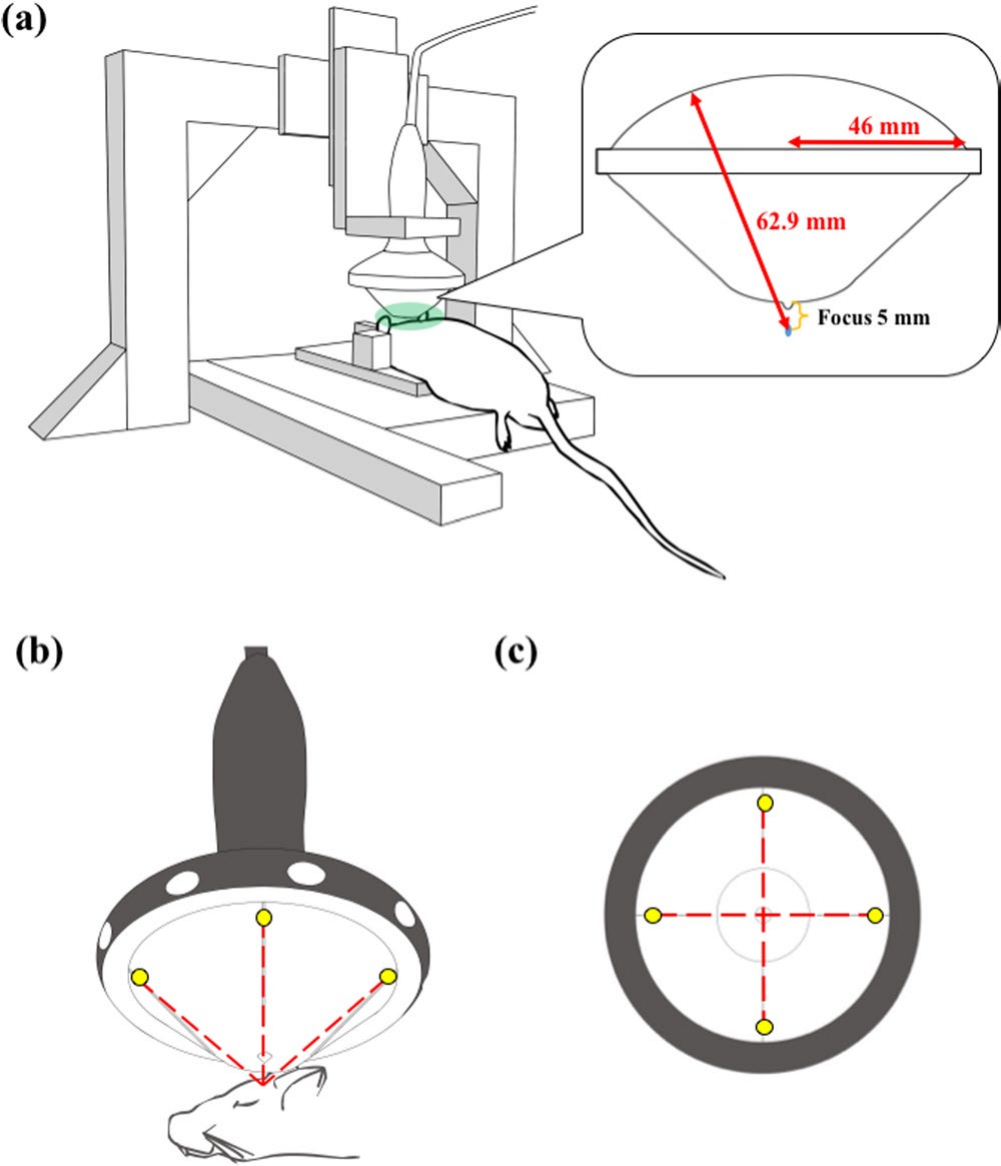
(**a**) The shockwave probe positioning platform and its experimental implementation; (**b**) experimental implementation of the shockwave probe and the head of the rat, wherein the yellow dots are the positioning lasers and the red dotted lines are the lasers trajectories; (**c**) bottom view of the shockwave probe, in which all experiments were processed with a shockwave focus positioned 5 mm from the interface of the rat’s scalp and the bottom of the gel pad.

Figure 1(a) shows the setup of the commercial shockwave device and the probe (F10, G4) positioning platform. The system was developed at the beginning of the proposed study to improve experiment repeatability and accuracy in applying shockwaves to the target region. A LabView-based computerized program was used to effectively decrease human error, with the accuracy of the positioning operational program reaching a precision of 0.1 mm (i.e. 2.5 mm ± 0.1 mm). Various distances from the scalp of each locating position were conducted with five independent samples (N = 5). Furthermore, 4 positioning lasers were situated on the outer rim of the shockwave probe (as shown in Fig. 1(b and c)) to enhance positioning mechanism and accuracy.

The concave shockwave probe (46 mm in radius and 62.9 mm in radius of curvature) was coupled with a gel pad to ensure the focus of the shockwave probe was 5 mm from the bottom of the gel pad. The shockwave probe, together with the gel pad, was positioned on top of the rat scalp to produce a focus 5 mm beneath the scalp surface and 3 mm caudal, 3 mm right or left of the bregma of the rat’s skull. Ultrasound gel was spread at the interfaces between the shockwave probe and the gel pad, and also between the bottom of the gel pad and the rat’s scalp.

### Shockwave induced BBB opening, its reversibility and ultrasound contrast agent (UCA) effect

Evans blue (EB) was used as an indicator to show the location and extent of BBB opening in rat brains since EB cannot penetrate an intact BBB. Immediately after the injection of 0.5 ml of 3% EB via the rats’ tail vein, 100 shockwave iterations were applied (intensity level 5, pulse repetition frequency (PRF) 5 Hz, N = 8). The following process was modified from a previous study^27^. The original two hour interval time for euthanization was extended to three hours. Three hours after the shockwave application, the rats were sacrificed and the brains were harvested for histological analysis^27^. The intensity levels for each parameter are shown in Table 1.

**Table 1:** Major shockwave parameters of the intensity levels.

Intensity level	0.1	1	2	3	4	5	10	20
Negative peak pressure (MPa)	−4.2	−7.3	−7.92	−8.54	−9.17	−9.79	−12.9	−18.7
Positive peak pressure (MPa)	5.4	11.8	13.66	15.51	17.37	19.22	28.5	77.7
Energy flux density (Total) (mJ/mm^2^)	0.03	0.1	0.13	0.16	0.18	0.21	0.35	0.82

To evaluate the performance of shockwave applications, the successful (visible) BBB opening rate is defined as number of rats with BBB opening after shockwave treatment over the total number of rats receiving shockwave treatment. To define BBB opening, the Evans blue stained area of the histology sections were further analyzed using a color histogram of Image J. The delta blue and delta red in the proposed study were the differences in pixels of blue and red color in RGB-image system that were used to evaluate the staining level of Evans blue and the red blood cell extravasation level between the shockwave applied side and untreated side. Both of them were assessed using student’s t-test and one-way ANOVA and post-hoc analysis, wherein p < 0.05 for the delta blue between the shockwave applied side and untreated side.

To confirm the reversibility of the shockwave-induced BBB opening, 0.5 ml of 3% EB was injected via the rats’ tail vein 24 hrs after applying 100 shockwave iterations with all other conditions remaining constant (N = 5). The rats were then sacrificed for brain sectioning 3 hours after the EB injection.

Traditionally, ultrasound contrast agent (UCA) is used together with HIFU to induce BBB opening in the brain by enhancing cavitation^28,29^. The effect of UCA on shockwaves was investigated by adding various concentrations of SonoVue UCA from 2.5 × 10^2^ to 2.5 × 10^8^ microbubbles/kg body weight (the clinical concentration is 3–15 × 10^6^ microbubbles/kg) through the tail vein immediately before shockwave treatment (N = 5).

### Gene transfection and *in vivo* optical imaging system analysis

The proposed gene transfection was processed by modifying the steps in the section of shockwave induced BBB opening, its reversibility and UCA effect, wherein 100 μg of pCI-neo-Luc+ was pre-dissolved in 50 μl of 150 mM NaCl and then injected into the rat’s tail vein prior to Evans blue injection. Transfection was then carried out using 200 shockwave iterations (intensity level 5, PRF 5 Hz, N = 5). After 72 hrs, 1 ml of the substrate (40 mg/ml D-Luciferin Firefly) was injected into each rat’s tail vein and allowed to react for 10 min. The anesthetized rats were sacrificed and their brains were placed into the *in vivo* optical imaging system (IVIS) scanning chamber to measure gene transfection performance. All IVIS data in the proposed research were analyzed using Living Image 3.1 (Caliper Life Sciences, Waltham, US).

### Histology sections

To evaluate the extent of tissue damage caused by various degrees of shockwave intensity and duration and by ultrasound contrast agent (SonoVue) concentrations, the experimental procedure described in the section of shockwave induced BBB opening, its reversibility and UCA effect was repeated and the brains were sliced using brain matrices immediately following shockwave treatment. The brain fillets were then immersed in 10% formaldehyde solution for 24 hrs. Subsequently, the specimens were embedded in paraffin and subjected to H&E, TUNEL assay (for apoptosis), GFAP stain (for glial cells). Slides were analyzed using an Olympus BX51 microscope with the Spot microscope camera system and its software, SPOT advanced.

### Statistics

All data are expressed as mean ± standard deviation (SD) of at least five independent samples (N). All statistical evaluations were carried out with one-way ANOVA and post-hoc analysis. A p-value of less than 0.05 was considered significant.

## Results

### Localization of shockwave probe

**Figure 2:**
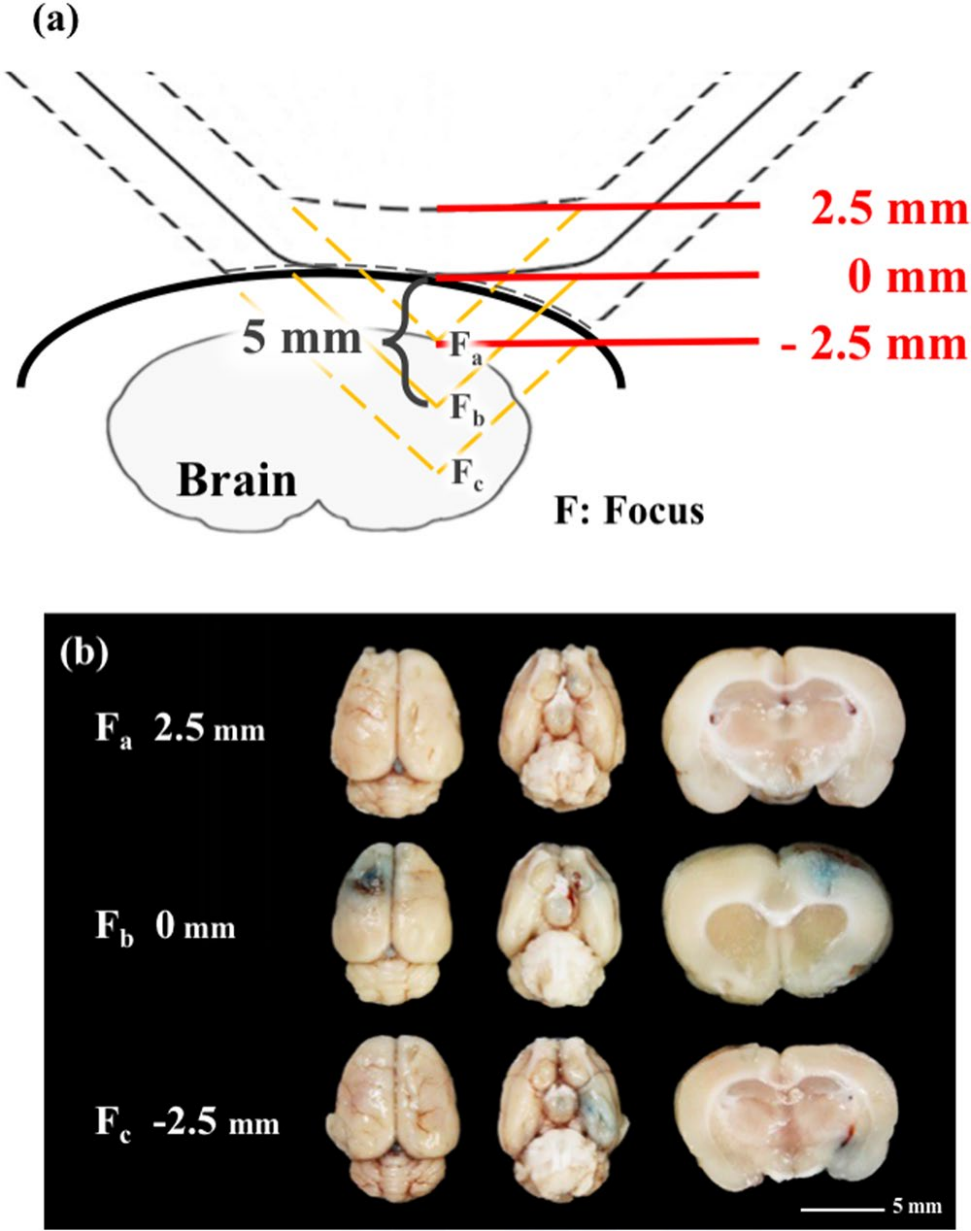
(**a**) Schematic of different penetration depths from the focus shockwave probe; (**b**) BBB opening at various distances from the scalp (2.5 mm, 0 mm, −2.5 mm). Shockwave treatment was carried out at an intensity level of 5 at PRF 5 Hz, in 500 iterations (N = 5). Scale bar was 5 mm.

As shown in Fig. 2(b), a BBB opening can be consistently produced at the focus of the shockwave probe (0 mm). As shown in Fig. 2(a), when the probe is vertically moved 2.5 mm away from the scalp surface, and the gap is filled with ultrasound gel, no BBB opening was found since the shockwave focus was above the brain region. On the other hand, when the shockwave probe was compressed 2.5 mm against the scalp (i.e., −2.5 mm), with the gel pad deformed, the opened regions moved downward to the lower part of the rat’s brain. Thus, the BBB opening regions can be precisely controlled in a rat brain, which has a volume approximately 1/600 of a human brain.

### Threshold of shockwave-induced BBB opening and reversibility

**Figure 3:**
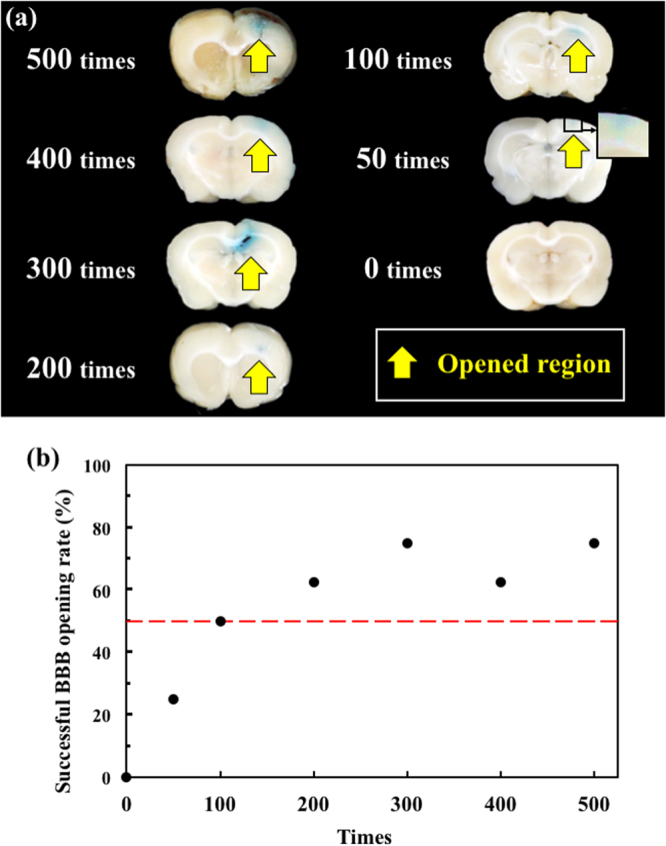
(**a**) Histology sections at 0, 50, 100, 200, 300, 400, and 500 shockwave treatment iterations; (**b**) successful BBB opening rate under 0, 50, 100, 200, 300, 400, and 500 shockwave treatment iterations. Shockwave treatment was conducted with an intensity level of 5 and PRF of 5 Hz. N = 8.

Figure 3(a) shows that the BBB opening region (the blue-stained area) could be repeatedly produced at the junction of the cortex and subcortical area of the rat’s brain. Based on Fig. 3(a) and Table 2, above the threshold, the blue-stained area and shade of the opening were not totally dependent on the number of shockwave treatments or the applied energy level. These results show that increasing the number of shockwave applications not only causes visible color changes and bleeding (as shown in the 0 mm top view of Fig. 2(b), and 300 times in Fig. 3(a)), but also results in larger standard deviation values. According to Fig. 3(b), at an intensity level 5, at least 100 shocks were needed to achieve a successful (visible) opening ratio, which is around 50%.

**Table 2:** Differences between red and blue colors of shock applied area as compared to the control area on histology sections.

Shocks	Δ Blue (Pixel)		Δ Red (Pixel)		Visible Bleeding (N)
	AVG	STDEV	AVG	STDEV	
500	30.5^*,¶^	27.0	64.5^*,¶,#^	51.1	4
400	39.8^*,¶^	58.2	55.1^*,¶^	59.1	2
300	40.5^*,¶^	31.9	69.8^*,¶,#^	41.9	2
200	23.5^*,¶^	17.4	56.0^*,¶^	36.4	0
100	22.3^*,¶^	16.6	26.0^*,¶^	15.1	0
50	7.8	9.2	10.8	9.1	0
0	3.9	2.5	7.3	5.4	0

*Significant difference of delta blue or red value to control (0 time).

^¶^Significant difference of delta blue or red value to 50-times group.

^#^Significant difference of delta red value to 100-times group.

As shown in Table 2, based on One-way ANOVA and post-hoc analysis for the p value of the difference on delta blue between the 0 and 50 times groups is 0.28, in which, delta blue was the different pixels of blue color in RGB-image system for evaluating the staining level of Evans blue between the shockwave applied side and untreated side. The p values of the remaining groups with more than 100 shockwaves iterations vs. the control (0 time) are less than 0.05. On the other hand, the p value of the differences for the delta red between the 100 and 200, 100 and 50 iteration groups are respectively 0.055 and 0.021, in which, delta red was the different pixels of red color in RGB-image system for evaluating the red blood cell extravasation level between the shockwave applied side and untreated side. The p values for comparing the remaining groups (i.e., 0, 200 to 500 iterations) against the 100 iterations group one is less than 0.05. Therefore, 100 shockwave iterations at intensity level 5 were selected as the threshold for shockwave-induced BBB opening.

**Figure 4:**
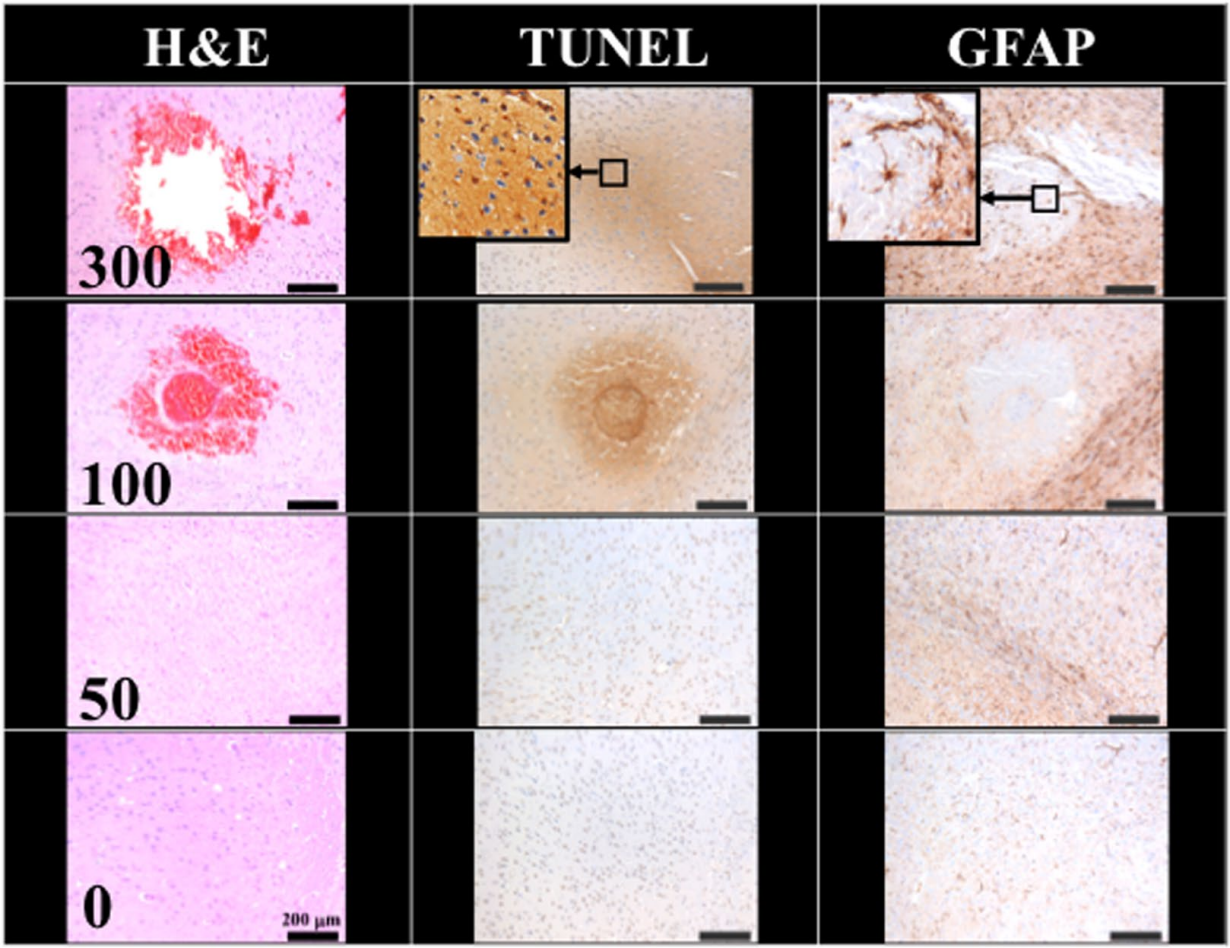
Histology sections of H&E, TUNEL, and GFAP stain for 0, 50, 100, 300 shocks with an intensity level of 5 and PRF 5 Hz. Scale bar was 200 μm. N = 8. Dashed circle indicates a transverse cut of a vessel.

Figure 4 compares the H&E stain, TUNEL, and GFAP stain, indicating the 100 and 300 shock groups showed around 0.005 cm^2^ and 0.015 cm^2^ red blood cell extravasation in the H&E stain; serious cell apoptosis was shown by the concentrated cell nucleus (dark brown particles) on the TUNEL assay, and prosperous astrogliosis was shown by rich astrocytes (brown star-shaped cells) on the GFAP stain. Moreover, the 100 and 300 shock groups respectively showed red blood cell (RBC) extravasation and vessel breakage. The red circle at the center of the H&E stain for the 100 shock group is probably a transverse cut of a vessel (dashed circle). RBC extravasation and tissue damage were both absent in the 50 shock group. Therefore, the threshold shockwave treatment for BBB opening was estimated to be between 50 to 100 shocks at an intensity level of 5 and PRF 5 Hz.

**Figure 5:**
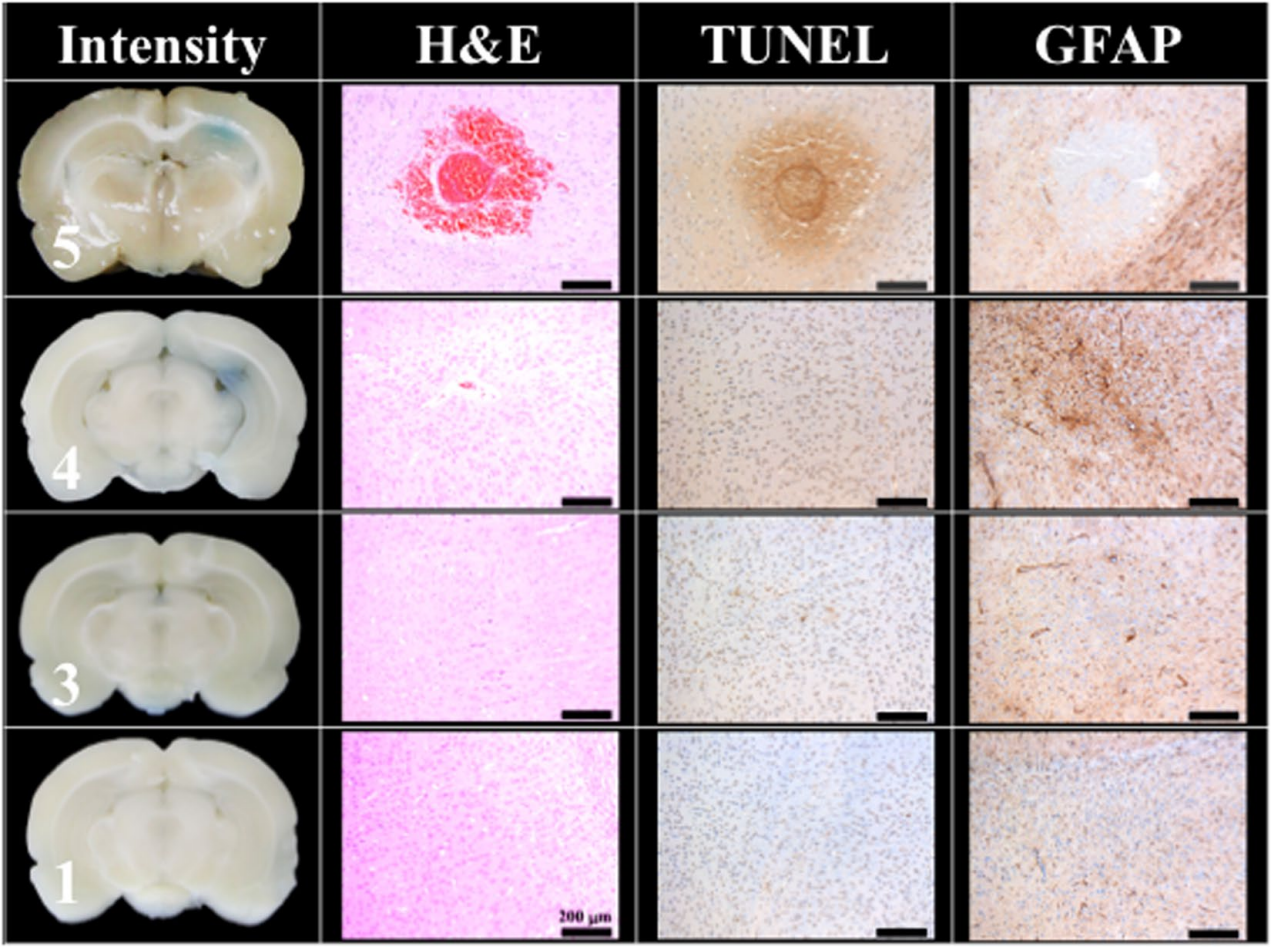
Histology sections of H&E, TUNEL, and GFAP stains for 100 shocks under intensity levels of 1, 3, 4, and 5. The PRF was set at 5 Hz. (Scale bar 200 μm, N = 5).

Figure 5 shows that the BBB could still be successfully opened at an intensity level of 4, with PRF 5 Hz, and 100 shocks, but the successful opening rate dropped to 20%, whereas the opening rate at intensity levels of 1 and 3 was 0%.

**Figure 6:**
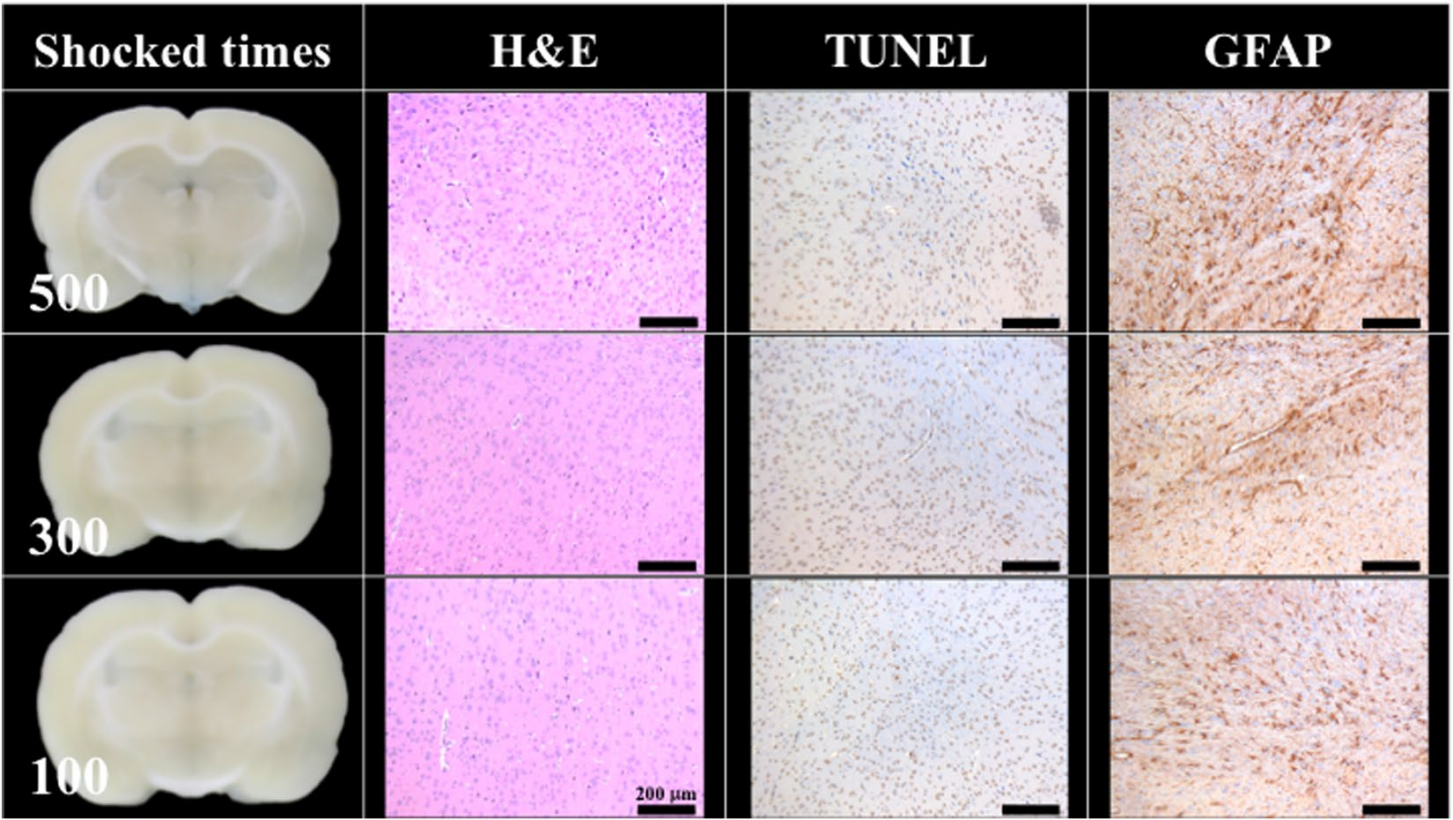
No BBB opening was found 24 hrs after 100, 300, 500 shocks at an intensity level of 5 and PRF 5 Hz. The indicator was 3% of Evans blue (pre-dissolved in 0.9% saline). Scale bar was 200 μm. N = 5.

Figure 6 shows the BBB opening reversibility. Twenty-four hrs after the completion of shockwave treatment, the tail vein infusion of EB could no longer stain the rat’s brain, indicating the BBB had closed. The area of concentrated astrocytes pointed out the region of shockwave-induced inflammation. However, no significant difference was observed among the 100, 300, and 500 shock groups (N = 5). Moreover, H&E stain and TUNEL assay failed to show any bleeding or extravasation.

### Shockwave-induced BBB opening in the presence of ultrasound contrast agent

**Figure 7:**
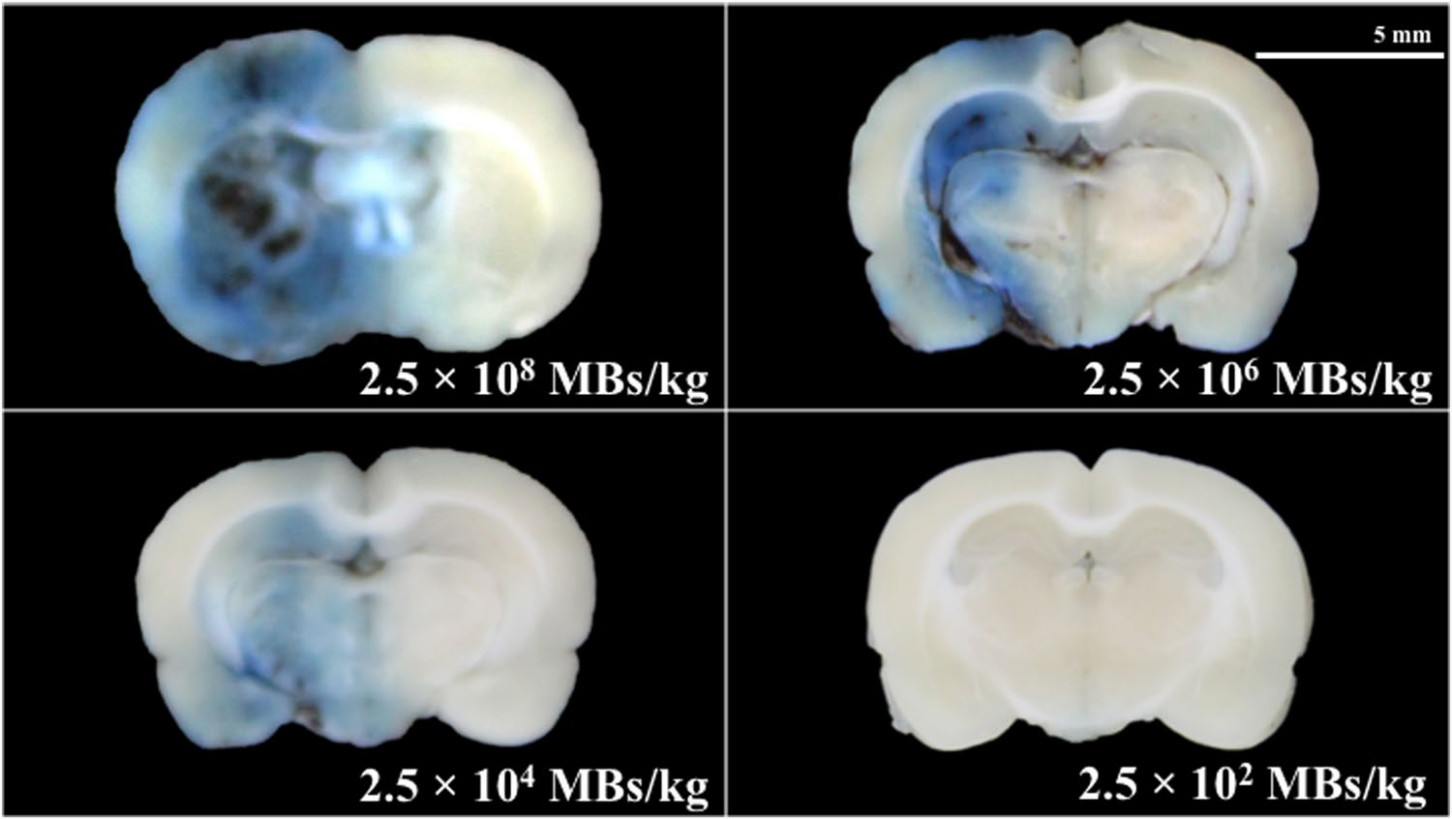
Histology sections after 50 shocks with different concentrations of UCA (SonoVue). Shockwaves were administered at an intensity level of 5, PRF 5 Hz. The indicator was 3% of Evans blue (pre-dissolved in 0.9% saline). Scale bar was 5 mm. N = 5. MBs: microbubbles.

Traditionally, UCA is used with HIFU to induce BBB opening. Figure 7 shows the effect of different concentrations of UCA on BBB opening via shockwave (N = 5). After the addition of 2.5 × 10^4^ MBs/kg of SonoVue UCA (125 nl/kg, approximately 1/400 of the clinical dosage), extensive BBB opening was achieved with an opening rate of 100% (data not shown). However, when the concentration exceeded 2.5 × 10^4^ MBs/kg (1/4 of the clinical dosage), extensive of bleeding and tissue damage occurred.

### Shockwave-based gene transfection

**Figure 8:**
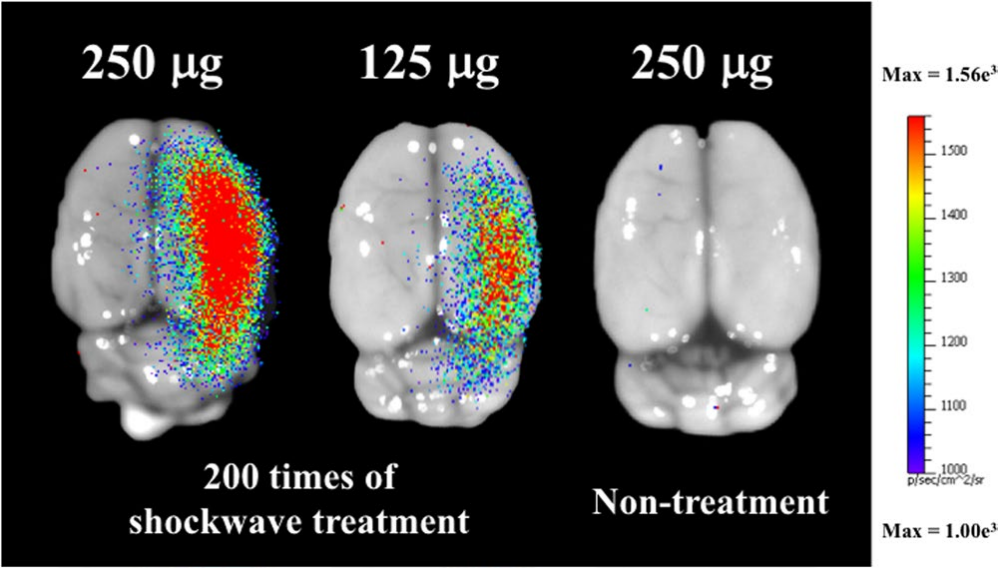
Luminescence image of shockwave-transfected rat brains with 250 and 125 μg of pCI-Neo-Luc+ (7187 bp, around 26591 Da) plasmids, and non-shocked control. Shockwave treatment was carried out 200 times at an intensity level of 5, PRF 5 Hz. N = 5.

To verify the performance of shockwave-induced brain gene transfection, shockwaves were applied after the tail vein injection of plasmid pCI-Neo-Luc+ (7187 bp, around 26591 Da) without UCA (N = 5). The luciferase luminescence shown in Fig. 8 and Table 3 confirm the successful transfection of plasmid DNA into the treated rat brains. As compared to the control group, the shockwave stimulus group showed significantly more luminescence, as well as a dose-dependent phenomenon between the 250 and 125 μg groups.

**Table 3:** LUC-FLUX of shockwave based transfection.

Brain side	250 mg		125 mg		250 mg wo/SW	
	Left	Right	Left	Right	Left	Right
AVG	875	3168	1044	2668	729	790
STDEV	115	318	91	136	16	14

## Discussion

Future clinical applications must minimize tissue damage induced during the BBB opening process. As shown in Fig. 3(a) and Table 2, the high variation between the shock times and the blue stained area and shade of the opening may be caused by the breakage of random vessels. According to the one-way ANOVA analysis results shown in Table 2, the p value of the difference on delta red and blue, they could be αured out the threshold of shockwave application under intensity level 5, PRF 5 Hz is 100 times.

As shown in Fig. 4, only a tiny amount of red blood cells extravasation occurred in the rat brain (0.005 cm^2^, only 0.3% of the slice area) with the intensity level 5, PRF 5 Hz and 100 shockwaves. As shown in Fig. 5, an intensity level of 4 or a reduced number of shocks (e.g., 50 times) produced successful BBB opening (visible EB), but with no detectable red blood cell extravasation.

Figure 4 also shows a small amount of red blood cell extravasation occurred after 100 and 300 shocks at intensity level 5 and PRF 5 Hz. However, after 24 hrs, this extravasation and the BBB had nearly completely recovered (Fig. 6). Only a small area of inflammatory response could be observed by GFAP stain. Therefore, based on the results of Figs 4 and 5, they probes the two important parameters (i.e. shock-times and intensity) of the proposed study, assesses the shockwave threshold range (100 shocks at an intensity level of 5 and PRF 5 Hz) and clarifies the red blood cells extravasation range (only 0.3% of the slice area).

**Figure 9:**
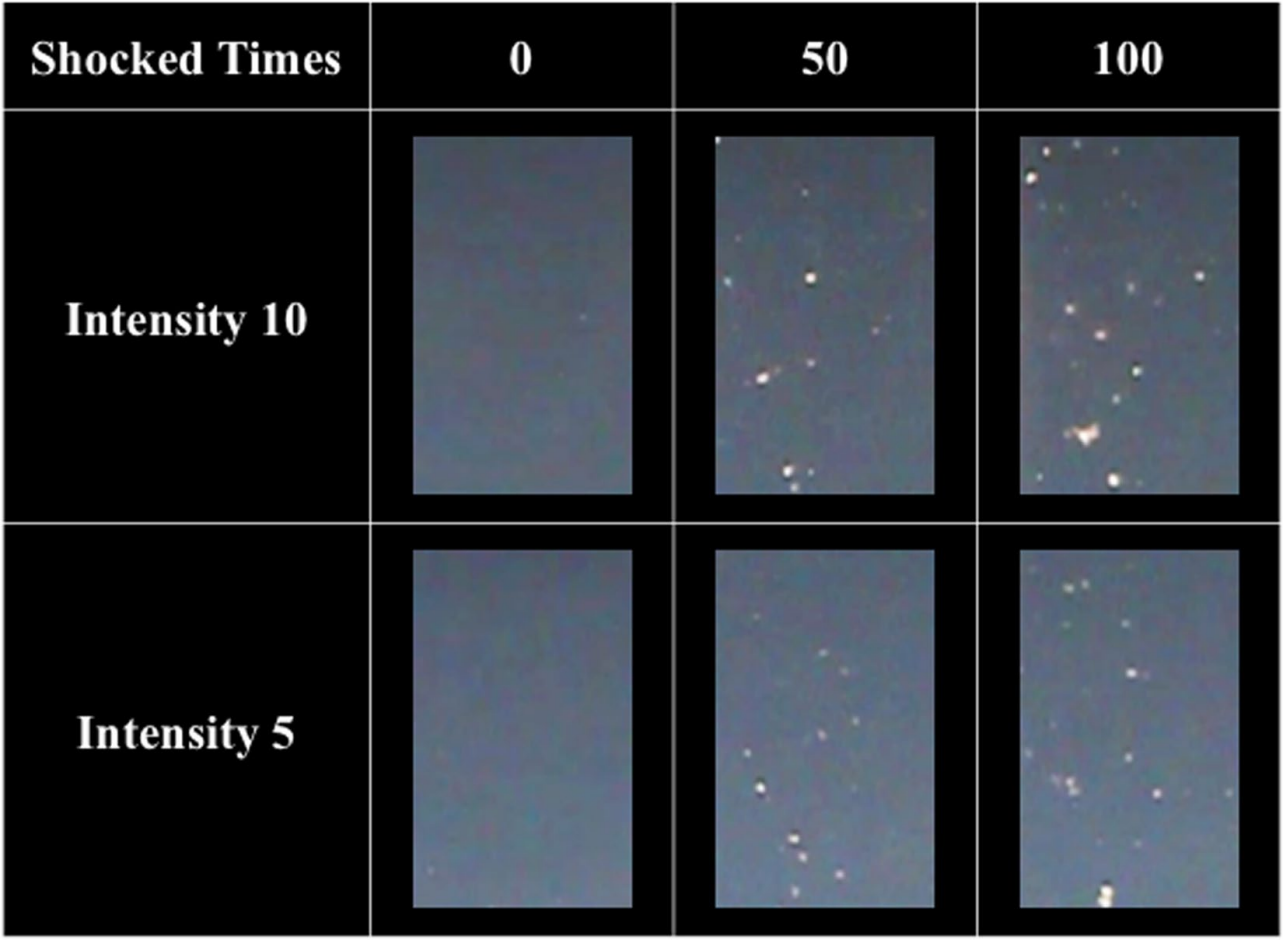
Bubbles generated after 0, 50, and 100 shocks at PRF 5 Hz and intensity levels of 10 and 5.

Figure 9 shows the bubbles generated by shockwaves in NIPAM hydrogel phantoms, wherein, the NIPAM hydrogel phantoms was made based on Sun’s study, which could demonstrate the shockwave induced cavitation phenomenon^30^. More and higher intensity shockwave treatment induced more and larger areas of bubbles in the phantoms, implying more cavitation activity leading to larger areas of BBB opening and tissue injury (i.e., red blood cells extravasation, apoptosis and glia cell infiltration). The extensive red blood cell extravasation produced by the addition of UCA is probably due to the high peak negative pressure of the shockwaves and strong cavitation effect in the rat brains (see Table 1 for detailed parameters)^24–26^.

Compared to the focal BBB opening using HIFU, the proposed shockwave-induced BBB opening method provides benefits such as obviating the need for UCA, shorter treatment time, flexible focal depth selections with gel pads, deeper penetration, and greater ease of crossing the skull with lower frequency shockwaves (Table 4). Moreover, the proposed study was conducted using a commercially available shockwave device, thus avoiding the expense of manufacturing a dedicated HIFU device. The focal area of the piezoelectric shockwave device is smaller than that of other existing shockwave devices, and therefore may be more suitable for CNS applications.

**Table 4:** Comparisons of key factors between HIFU and shockwave induced BBB opening.

Device	HIFU					UCA	
	Pressure (MPa)	Frequency (MHz)	PRF (Hz)	Burst length (ms)	Duration (sec)	Dose (Mega-MBs/kg)	Brand
Single element FUS transducer (Imasonic)^31^	0.75	1.50	5	6.56	300	2000	Self-manufactured
Therapy Imaging Probe System focused ultrasound transducer (Philips)^32^	0.50	1.00	100	10.00	600	400	Advanced Microbubble Laboratories
Single element FUS transducer (Imasonic)^33^	0.72	1.50	5	10.00	300	800	Self-manufactured
Lead zirconate titanate (DeL Piezo Specialties)^34^	0.39	0.55	1	10.00	120	200	Definity
H-107 spherical-segment FUS transducer (Sonic Concepts)^35^	0.60	0.50	2	10.00	120	250	Self-manufactured
H-107 spherical-segment FUS transducer (Sonic Concepts)^36^	0.60	0.50	2	10.00	120	125	Self-manufactured
RK100 focused ultrasound transducer (FUS Instruments Inc.)^37^	1.09	0.55	1	10.00	120	200	Definity
RK300 focused ultrasound transducer (FUS Instruments Inc.)^38^	0.55	1.00	1	10.00	120	6	Definity
**Device**	**Shockwave**					**UCA**	
	**Pressure** ^**+**^ **(MPa)**	**Pressure** ^**−**^ **(MPa)**	**Rise time (ns)**	**PRF (Hz)**	**Duration (sec)**	**Dose (Mega-MBs/kg)**	**Brand**
Piezowave (The current study)	17.37	−9.79	174	5	10	—	—

Based on the above findings, shockwave-induced BBB opening shows significant therapeutic potential for the treatment of CNS diseases. Increased endothelial permeability will allow drugs including antibiotics, chemotherapeutic agents, biologic agents and other medical substances with large molecular mass to cross the BBB, offering more choices for clinical medication and enhancing bioavailability in the CNS to achieve optimal therapeutic concentrations. For instance, focal BBB opening may facilitate chemotherapeutic agents entering the targeted area while sparing normal brain tissue. Meanwhile, the reversibility of shockwave-induced BBB opening reduces the possibility of opportunistic infections, and the healed endothelium protects the CNS system from lethal pathogens. Treatment of neurodegenerative diseases such as Parkinson’s disease may also benefit from shockwave-induced focal BBB opening, and the controllable focal BBB opening raises the possibility of regional gene therapy. Gene transfection into neurons in the substantia nigra has potential to restore neuron function, and is a promising method of disease-modifying therapy.

## Conclusions

This study presents a novel BBB-opening system consisting of a commercial piezoelectric shockwave device, an automatic motion stage, and a laser targeting system to perform precise and stable BBB-opening in rat brains. Different from a traditional high-intensity focused ultrasound (HIFU) device for BBB-opening (Table 5), the shockwave system emits higher pressure waves but avoids the use of ultrasound contrast agent (UCA), allowing simpler and improved control of cavitation-facilitated BBB opening, which may benefit future neuro-oncology and neurophamacology research and applications.

**Table 5:** Comparison of shockwave and HIFU induced BBB opening.

	Working frequency	Depth of penetration	Thermal effects	Focus	Commercial device
Shockwave	Low	Deep	Less	Flexible	Yes
HIFU	High	Low	High	Rigid	No
